# Supplementary material for: Online In-Tube Solid-Phase Microextraction Coupled with Liquid Chromatography–Tandem Mass Spectrometry for Automated Analysis of Four Sulfated Steroid Metabolites in Saliva Samples
Source: Molecules. 2022 May 18;27(10):3225. doi: 10.3390/molecules27103225 (PMC9145352; doi:10.3390/molecules27103225)
Supplement: Supplementary file 1 [file molecules-27-03225-s001.zip › molecules-1732715-supplementary.pdf]

Supplementary Materials

Online In-Tube Solid-Phase Microextraction Coupled with Liquid Chromatography–Tandem Mass Spectrometry for Automated Analysis of Four Sulfated Steroid Metabolites in Saliva Samples

Hiroyuki Kataoka * and Daiki Nakayama

School of Pharmacy, Shujitsu University, Nishigawara, Okayama 703-8516, Japan

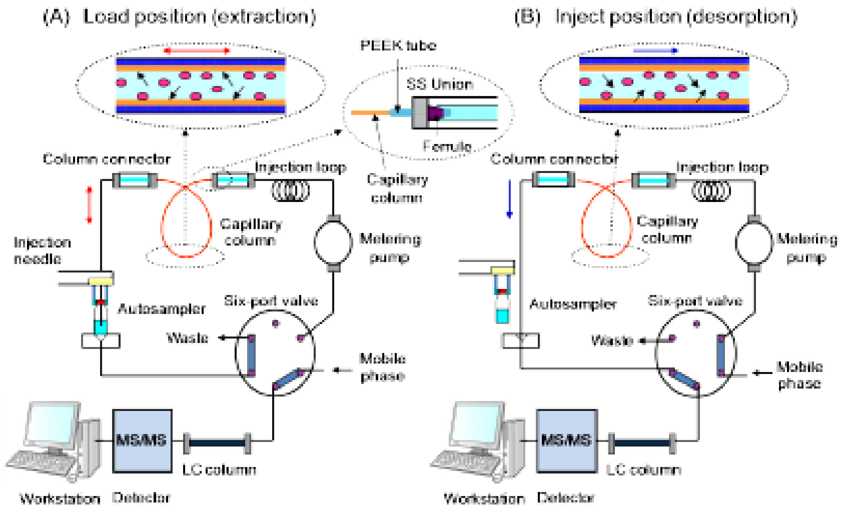

Figure S1. Schematic diagrams of the automated online IT-SPME/LC–MS/MS system.

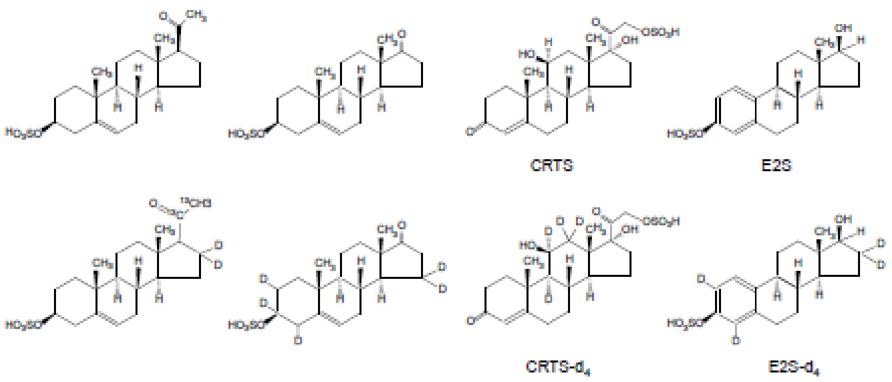

Figure S2. Structures of sulfated steroid metabolites and their respective stable isotope-labeled compounds as internal standards.

Table S1. Program for the IT-SPME process.

Sequence	Event	Switching valve	Vial	Draw/ejection		
				Cycle[1]	Volume (µL)	Speed ($\mu L\ min^{-1}$)
1	Conditioning of the capillary	Load	MeOH	D/E (2)	40	200
2	Drawing of air into the capillary	Load	Empty	D (1)	50	200
3	Conditioning of the capillary	Load	Water	D/E (2)	40	200
4	Extraction of analytes into the capillary	Load	Sample	D/E (30)	40	200
5	Needle washing	Load	MeOH	D/E (1)	2	200
6	Desorption of analytes from the capillary	Inject	–	–	–	–
7	HPLC separation of analytes and return to sequence 1	Load	–	–	–	–

[1] D: draw, E: ejection.

Table S2. MRM transitions and setting parameters for sulfated steroid metabolites and their stable isotope-labeled compounds.

TSNA	RT[1] (min)	Mass transition (m/z)	Dwell time (msec)	DP[2] (V)	EP[3] (V)	CE[4] (V)	CXP[5] (V)
PREGS	3.9	395.0 →96.8	125	-65	-8	-65	-10
DHEAS	2.7	367.0 →97.0	125	-60	-8	-60	-5
CRTS	2.1	441.2 →97.0	125	-65	-8	-65	-15
E2S	2.3	351.3 →80.1	125	-65	-8	-60	-5
PREGS-$^{13}C_2$-d_2	3.9	399.0 →96.8	125	-65	-8	-65	-10
DHEAS-d_5	2.7	372.4 →97.4	125	-60	-8	-60	-5
CRTS-d_4	2.1	445.2 →96.8	125	-65	-8	-65	-15
E2S-d_4	2.3	354.7 →80.0	125	-65	-8	-60	-5

[1] Retention time (min)
[2] Declustering potential (V)
[3] Entrance potential (V)
[4] Collision energy (V)
[5] Collision cell exit potential (V)
